# Recruitment and retention strategies in mental health trials – A systematic review

**DOI:** 10.1371/journal.pone.0203127

**Published:** 2018-08-29

**Authors:** Yifeng Liu, Emma Pencheon, Rachael Maree Hunter, Joanna Moncrieff, Nick Freemantle

**Affiliations:** 1 Research Department of Primary Care and Population Health, University College London, London, United Kingdom; 2 Chelsea and Westminster NHS Foundation Trust, London, United Kingdom; 3 Division of Psychiatry, University College London, London, United Kingdom; 4 Institute of Clinical Trials and Methodology, University College London, London, United Kingdom; Ulster University, UNITED KINGDOM

## Abstract

**Background:**

Recruitment and retention challenges are very common in mental health randomised trials. Investigators utilise different methods to improve recruitment or retention. However, evidence of the effectiveness and efficiency of these strategies in mental health has not been synthesised. This systematic review is to investigate and assess the effectiveness and cost-effectiveness of different strategies to improve recruitment and retention in mental health randomised trials.

**Methods and materials:**

MEDLINE, EMBASE, the Cochrane Methodology Register and PsycINFO were searched from beginning of record up to July 2016. Randomised trials involving participants with mental health problems which compared different strategies for recruitment or retention were selected. Two authors independently screened identified studies for eligibility.

**Results:**

A total of 5,157 citations were identified. Thirteen articles were included, 11 on recruitment and 2 on retention. Three randomised controlled trials compared different recruitment strategies, none of which found statistically significant differences between the interventional recruitment strategies and the routine recruitment methods. Retrospective comparisons of recruitment methods showed that non-web-based advertisement and recruitment by clinical research staff each have advantages in efficiency. Web-based adverts had the lowest cost per person recruited (£13.41 per person recruited). Specialised care referral cost £183.24 per person, non-web-based adverts cost £372.03 per patient and recruitment via primary care cost £407.65 for each patient. Financial incentives, abridged questionnaires and pre-notification had a positive effect on retention rates.

**Conclusion:**

The recruitment studies included showed differences in strategies, clinical settings, mental health conditions and study design. It is difficult to assess the overall effectiveness of any particular recruitment strategy as some strategies that worked well for a particular population may not work as well for others. Paying attention to the accessibility of information and consent materials may help improve recruitment. More research in this area is needed given its important implications.

## 1. Introduction

Recruitment and retention in randomised clinical trials are challenging. [1] Delayed recruitment can give rise to a series of issues, such as additional costs or the need for an extension of the study period. Inadequate or ineffective recruitment may often result in reduced power of the study or even premature termination of a trial, and can lead to the trial being unable to answer important clinical questions. [2] Loss to follow-up and patient dropouts can also result in reduced study power, hindering the ability to detect potential differences between trial arms should they exist, as well as undermining the internal validity of the trial. Although an increasing amount of research has contributed to dealing with missing data in clinical trials, the risk of bias due to missing data cannot be avoided through the application of statistical techniques for missingness, such as multiple imputation, as these techniques require additional assumptions which may not be valid. It has been suggested that less than 5% loss to follow-up may lead to an unimportant level of bias, while 20% or greater loss to follow-up poses a substantial threat to a trial’s internal validity. [3] Some modern trials aim to reduce this risk by increasing the sample size by 20%, which addresses precision but not internal validity, and poses a further challenge to recruitment.

Although generally considered the gold standard of clinical research, randomised controlled trials often fail to recruit enough participants, particularly in the area of mental health. [4] Extensive collaboration is often required between researchers, patients, clinical professionals and institutions, and each party in a clinical trial has its unique expectations and concerns towards the trial. [4] Concerns from clinicians about mental health patients’ vulnerability and reduced decision-making ability may make recruitment difficult. [5] For patients particularly, doubts about getting involved in a trial primarily centre around their own health, that is, how they could benefit, or what potential issues they might be faced with in the treatment being investigated. During the consent process, where potential participants are introduced to the trial’s protocol, they could be put off by aspects of the study which may appear inconvenient, abstruse or irrelevant. [4]

The fundamental biological aetiology for some mental health conditions is still not well understood, and often the effects of psychiatric treatments are small and uncertain. Hence there may be scepticism that new treatments will be very helpful, which might make psychiatric trials less appealing. [6–8] High placebo response rates also highlight the importance of randomised trials in providing unbiased estimates of treatment effects. Patients with mental health problems often still consider their conditions as stigmatised (sadly often for good reason) and conceal their condition and treatment from public attention. Also, for some mental illnesses, there are ethical concerns when involving patients who are at high risk or have a history of aggression or self-harm. [9] These concerns make recruitment to mental health clinical trials challenging.

Retention is another pivotal component to a trial’s scientific success as it is key to a trial’s validity. Attrition may happen in drug trials because of side effects of the medication. Some patients may experience deterioration of their health during the follow-up period, making them reluctant to continue with treatment or the trial. In trials of complex interventions, such as cognitive behavioural therapy or early supported discharge, the absence of blinding in the control arm means that the participants know that they have not received the intervention, which may reduce engagement with trial follow-up or increase the risk of drop-out. It has been suggested that high drop-out rates are associated with larger sample sizes in antipsychotic trials, more specifically trials with multi-centre design. [10] However in modern trials, the sample size required often necessitates a multi-centre design as a single site would not provide enough participants and may not provide sufficiently generalisable results. This requirement for a multi-centre design might result in retention issues.

Previous systematic reviews by Treweek *et al*. and Brueton *et al*. investigated the efficacy of different strategies to improve recruitment and retention to randomised trials. [11,12] However evidence in the mental health trial population remains sparse. The review by Treweek *et al*. summarised the efficacy of different strategies to improve recruitment into randomised trials, but only included 3 eligible studies in mental health. No mental health studies were included in the systematic review by Brueton *et al*., which investigated the efficacy of different strategies to improve retention. Treweek *et al*. found that open trial design, and telephone reminders to people who do not respond to postal invitations may improve recruitment, whereas bespoke participant information materials helped little in recruitment.[13] Offering a small financial incentive for completing follow-up questionnaires appeared to help retain patients in the trials, as suggested by Brueton *et al*. [12] An increasing number of studies employ the use of a “study within a trial” (SWAT) method to assess the impact of technical or design innovations on a trial’s efficiency.[14] To date, most different recruitment strategies are usually employed in an *ad hoc* manner. Evidence on comparing recruitment strategies retrospectively and observationally can also provide some insight before SWATs are planned.

The aim of this review is to evaluate the evidence base for strategies to improve the recruitment and retention of patients to clinical trials in mental health. A secondary aim was to evaluate the cost-effectiveness of different recruitment and retention strategies, reported as the cost per patient recruited, or cost per patient retained.

## 2. Methods

### 2.1 Criteria for considering studies for this review

#### 2.1.1 Types of studies

Two review authors independently screened titles and abstracts and any disagreements in selection were resolved through discussion. Studies that used randomised or observational methods to compare different recruitment strategies designed to recruit participants to randomised controlled trials of interventions for mental health problems were considered. Embedded randomised studies of different recruitment strategies were identified, but given the small number of such studies, we also included randomised controlled trials (RCTs) of mental health interventions which reported the effectiveness a range of strategies used in recruitment retrospectively (e.g. without randomising to different recruitment strategies). For retention, randomised trials of different retention strategies that were embedded in a randomised clinical trial (host trial), or within epidemiological studies such as cross-sectional surveys were included. A full description of the study protocol is available in S1 File.

#### 2.1.2 Types of data

Studies comparing recruitment or retention that involved adult participants with mental health problems, regardless of gender, ethnicity or geographic location, were included. Of particular interest were trials including patients with serious mental illnesses (SMI), such as schizophrenia, but given the expectation of finding only a small number of studies involving these patients, the criteria were broadened to include common mental health problems such as depression and anxiety. Dementia and other organic mental health conditions were excluded, given the different context in which these trials are likely to be conducted. Studies on substance misuse were also excluded as this group of patients is likely to present different recruitment and retention challenges. Studies which did not report outcomes on recruitment or retention strategies for RCTs, studies in which mental illness was comorbid with other physical medical conditions (e.g. cardiovascular disease), and studies not involving adults (e.g. children or adolescents) were also excluded.

#### 2.1.3 Types of methods

Strategies aimed at enhancing recruitment and retention included, but were not limited to:

· Incentives for either or both of patients and clinicians
· Advertising
· Periodic phone call follow-up
· Mailshots and newsletters
· Customised or optimised consent materials
· Amendments to protocol
· Presentations to appropriate groups
· Presentations at conferences
· Trial material customised to specific sites
· Resource manual for recruiters

### 2.2 Types of outcome measures

#### 2.2.1 Primary outcome

For recruitment, the main outcomes of interest were the type of strategies employed in different studies and the number of patients recruited using each individual strategy. We also extracted data on how many potential participants were approached, if available, using each different strategy in each study. For studies comparing different retention strategies, the primary outcome was ‘response’, defined as the percentage of participants who were successfully engaged in follow-up assessments via each strategy out of the total number of people initially randomised to that strategy.

#### 2.2.2 Secondary outcomes

We were also interested in the cost of each patient recruited/retained through a specific strategy (if any mentioned), namely, the cost-effectiveness of each strategy, defined as the mean cost per patient recruited or mean cost per patient retained, respectively.

### 2.3 Search strategy

We designed a search method for identifying published randomised trials that focused on improving recruitment and retention in mental health randomised trials. We did not apply any language restrictions apart from English language abstracts.

The search method was comprised of 4 components, each of which included both free-text terms and subject headings. The Boolean operator OR combined terms related to enhancing recruitment and improving retention. This was then combined using the AND operator with terms related to mental health conditions and randomised controlled trials. A brief search strategy is described as follows:

(informed consent OR recruit OR particip) OR (retention OR attrit OR retain)

AND

Randomi#ed controlled trials

AND

Mental health condition filters

Electronic databases searched included:

· MEDLINE, Ovid (1946 to date of search, searched on 28 July 2016);
· EMBASE, Ovid (1980 to date of search, searched on 28 July 2016);
· PsycINFO, Ovid (1806 to date of search, searched on 28 July 2016);
· Cochrane Methodology Review Group Specialised Register (CMR) (from inception until July 2012, searched on 28 July 2016).

The full search strategies for all of the 4 databases are included in S1 File.

### 2.4 Data extraction and analysis

#### 2.4.1 Data extraction

Two reviewers extracted data from eligible studies. Data extracted for the recruitment trials and their corresponding host trials included:

- For host trials: country, disease area, design, sample size, setting, primary outcomes, funding body;
- For embedded randomised recruitment trials: strategies to which participants were randomised, number of participants in each arm who were recruited to the host trial;
- For studies that compared recruitment strategies retrospectively: strategies used for recruitment, number of participants recruited and approached via each strategy.

For retention trials and their host trials, data extracted included:

- For host trials: country, disease area, design, sample size, setting, primary outcomes, follow up period, funding body;
- For retention trials: strategies to improve retention; retention rates for each strategy.

#### 2.4.2 Assessment of risk of bias

We used the Critical Appraisal Checklist for Randomised Controlled Trials developed by the Joanna Briggs Institute (JBI) for the assessment of risk of bias for the eligible studies, and calculated the overall score based on the number of items checked for each assessment. Details of the risk of bias assessments are included in S2 File.

#### 2.4.3 Data analysis

For randomised comparative studies, we used relative risk to describe the effect of each recruitment strategy. Non-randomised studies were categorised according to similarity of strategies, for instance, by combining optimised consent materials and incentives. We ranked the strategies based on the numbers of patients recruited and identified strategies that recruited most participants in each study. We also calculated the total number of patients randomised through each recruitment strategy for recruitment studies and the number of responses in each retention strategy for retention studies. Cost-effectiveness of strategies where cost data were available was measured by average cost per patient randomised or average cost per response, respectively. Cost information was first converted to the equivalent monetary value in 2016 using relevant inflation rates of the study country, and subsequently into GBP based on average exchange rates between each currency and GBP in 2016. (http://www.ukforex.co.uk/forex-tools/historical-rate-tools/yearly-average-rates). The average cost-effectiveness of each category of recruitment strategy was calculated using a weighted average approach, where the mean costs were weighted by the sample size.

For studies where cost data were not reported, we extracted relevant information on the processes and generated the cost using available reference cost information (from e.g. Personal Social Services Research Unit). Given the considerable uncertainty in this approach due to insufficient information on resources used, we also performed sensitivity analysis under different scenarios. For instance, the cost of recruiting using health care providers, or research staff, depends on the number of hours spent on recruitment. The assumptions made were: part-time (e.g. 3 hours/day) versus full-time (e.g. 7.5 hours/day). The costing mainly employed a bottom-up approach from a UK NHS/personal social services (PSS) perspective. The unit cost of each component mentioned during the recruitment process was multiplied by the recruitment duration, or study duration otherwise, before all the relevant cost components were summed to make the total recruitment cost. The cost-effectiveness of each strategy was obtained using the total cost divided by the total number of participants for each strategy.

## 3. Results

### 3.1 Results of the search

**Fig 1:**
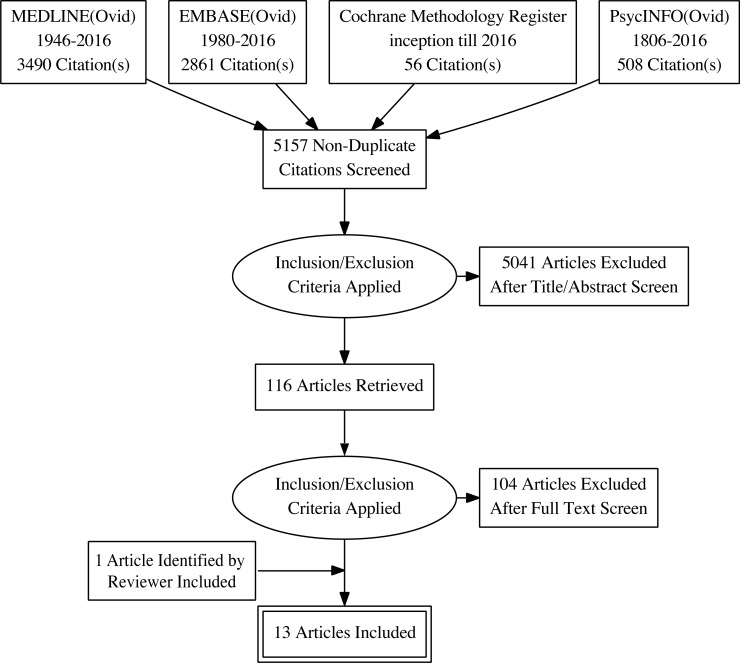
A PRISMA flow chart of the article selection process (generated from http://prisma.thetacollaborative.ca/).

We identified 5157 abstracts, titles and other records from electronic databases from inception of records until July 2016. Of these, 116 were identified for full-text screening and 12 were found to be eligible for inclusion. One additional study (Hughes-Morley 2016) identified by one of the reviewers of this article was also included. (Fig 1) Out of the 13 included studies, 11 are studies on recruitment strategies in the mental health clinical trial context, and 2 focused on retention. Five out of 13 were randomised comparative trials of different recruitment or retention strategies, and 8 were observational comparisons of recruitment strategies embedded within a randomised trial.

### 3.2 Recruitment strategies

#### 3.2.1 Characteristics of the included studies

Table 1 describes the characteristics of the studies included which looked at recruitment strategies. Overall, three studies employed a randomised design for comparing recruitment strategies (Man 2015, Jeste 2009 and Hughes-Morley 2016). The other studies compared different recruitment strategies retrospectively without randomisation.

**Table 1:** Summary of the characteristics of included studies on recruitment strategies.

Study ID	Trial design & intervention	Method of recruitment strategy comparison	Sample size (N)^1^	Study duration	Recruitment strategies	No. Patients recruited/No. Patients approached or where contact was attempted	Country
**Woolhouse 2014 [15]**	RCT of mindfulness vs TAU in women of depression, anxiety or stress	retrospective	32	6 weeks	a. researcher recruiting at clinic waiting room	14/50	Australia
					b. mailed-out brochures	16/2500	
					c. recruitment via physiotherapy and childbirth education classes	2	
**Krusche 2014 [16]**	RCT of mindfulness-based CBT vs TAU in preventing relapse in people with recurrent depression conducted in primary care	retrospective	153	8 weeks	a. word of mouth	16/46	UK
					b. information from charity	2/8	
					c. posters	30/123	
					d. web-based adverts	37/300	
					e. mental health care referral	8/32	
					f. radio adverts	26/412	
					g. GP referral	18/116	
					h. bus adverts	2/4	
					i. newspaper adverts	11/101	
					j. exhibition	3/11	
**Morgan 2013 [17]**	RCT of email delivered self-help health promotion intervention for adults with subthreshold depression symptoms to prevent depression (patients were screened online using PHQ-9)	retrospective	1699	6 weeks	a. Google advertising	755	Australia
					b. Facebook adverts	35	
					c. online forums	unknown^2^	
					d. links from mental health websites	unknown	
					e. online community noticeboards	unknown	
					f. group emails	unknown	
**Man 2015 [18]**	RCT of a telephone support and computer-based self-management intervention vs. usual care in patients with depression in primary care	RCT	60	12 months	a. optimised written patient information material	43/682	UK
					b. original patient information material	27/682	
**Rollman 2008 [19]**	RCT of telephone-based collaborative care for treating patients with DSM-IV panic and anxiety disorders	retrospective	369	Not reported	a. electronic medical record reminder to primary care clinicians to approach eligible patients	176/794	US
					b. waiting room recruitment by research staff	193/8095	
**Jeste 2009 [20]**	Hypothetical RCT of a cognition-enhancing drug vs. placebo in patients with DSM-IV schizophrenia	RCT	248	14 weeks	a. multimedia enhanced consent procedure	31/62	US
					b. ordinary consent procedure	29/66	
**Daley 2007 [21]**	RCT of an exercise intervention for women with postnatal depression	retrospective	38	12 weeks	a. recruitment via GP	19/96	UK
					b. recruitment via specialised “mother and baby” unit	9/28	
					c. recruitment by health visitors	7/10	
					d. self-referral	3/4	
**Le 2008 [22]**	RCT of an antenatal psycho-educational group intervention to prevent postpartum depression in patients with high risk^3^	retrospective	310	8 weeks	a. recruitment by community health centre staff	276/553	US
					b. recruitment by clinical research staff at hospital-based clinic	34/1349	
**Debar 2009 [23]**	RCT of a cognitive behavioural therapy-based guided self-help program on patients with DSM-IV Binge Eating Disorder	retrospective	249	not reported	a. mail invitation to comprehensive Eating Disorders Examination (EDE) assessment, $5 incentive for completing online screening questionnaire and $50 for baseline assessment b. mail invitation to abbreviated EDE assessment + telephone interview, $25 for baseline assessment (no payment for screening)		US
						70/11984	
						154/20810	
					c. self-referral	25/87	
**Schlernit-zauer 1998 [24]**	RCT of nortriptyline and interpersonal psychotherapy in elderly patients (age ≥ 65) with bereavement-related major depression (screened using HAM-D scale).	retrospective	65	Not specified	a. adverts	35/194	US
					b. obituary letter	9/99	
					c. acquaintance/friend	9/54	
					d. outpatient/in-house psychiatric referral	7/47	
					e. non-specific resources	2/20	
					f. non-mental health physicians	3/11	
					g. letters sent to medical community/health professionals	0/7	
					h. inpatient psychiatric referral	0/5	
					i. private mental health practitioner	0/3	
					j. other mental health facilities	0/1	
**Hughes-Morley 2016 [25]**	EQUIP host trial–clustered RCT of a new user led training package to increase user and carer involvement in care planning for patients with a diagnosis of severe mental illness under community mental health teams	RCT and Retrospective^4^	480	30 months	a. leaflet sent to advertise patient and public involvement in research (PPIR)	216/5382	UK
					b. control (without leaflet)	148/2800	
					c. leaflet sent to advertise PPIR + telephone follow up for non-responders	129/4988	
					d. control + telephone follow up for non-responders	92/2580	

Notes

1. For randomised recruitment trials, N = sample size of its host trial. For non-randomised studies, we assume that the sample size is the sum of number of patients recruited via each strategy.

2. According to the Morgan (2013), there was a total number of 94,808 approaches made in the study.

3. According to Le (2008), high risk = Epidemiologic Studies Depression Scale (CES-D) ≥ 16; all patients were self-reported.

4. In Hughes-Morley (2016), patients who were enrolled during telephone follow up (strategy c & d) were not included in the primary outcome as this was not the intervention for which this trial was designed to find evidence.

Four studies were carried out and funded in the UK, 5 in the US and 2 in Australia. One study involved recruitment to a preventive programme for depression, and one involved a relapse prevention trial in women with a history of post-partum depression. Two of the studies was conducted with people with severe mental illnesses. Five were carried out in a primary care setting. Four involved female participants only. Except for one RCT which was a study of recruitment to a hypothetical trial, the studies involved recruitment to randomised trials involving a range of interventions including mindfulness cognitive behavioural therapy (CBT), health promotion via email, telehealth intervention, exercise, antidepressants, interpersonal therapy and psycho-education. A brief description of the included studies is available in S1 Supplementary.

#### 3.2.2 Randomised comparative studies

Of the included studies, Jeste 2009, Man 2015 and Hughes-Morley 2016 used a randomised approach to compare alternative recruitment strategies. (Table 2) Jeste *et al*. compared a multimedia consent process using a DVD to present key information from the consent form, with routine consent procedure plus a 10 min ‘control’ DVD giving general information about research. Man *et al*. used an ‘optimised’ version of the trial information sheet, with contrasting colour, larger fonts, bulleted lists, and accessible wording, compared with the original 8-page A5 patient information booklet. Hughes-Morley *et al*. investigated the impact of a strategy of providing a leaflet describing the patient and public involvement (PPI) in the trial on recruitment of people who had a diagnosis of severe mental illnesses. Using multimedia during the consent process did not significantly improve recruitment in patients with schizophrenia, whereas optimised written patient information material was superior to non-optimised information for recruitment of patients with depression in primary care, but this result may have occurred by chance. Finally, offering information on PPI collaboration on the trial was not found to have a positive impact on trial recruitment.

**Table 2:** Summary of randomised comparative studies on recruitment strategies.

Study ID	Strategy comparison (intervention vs. control)	No. Patients recruited / No. Patients attempted (intervention)	No. Patients recruited / No. Patients attempted (control)	Relative Risk
**Jeste 2009**	DVD multimedia consent with key information from consent form vs. routine consent procedure + 10 min control DVD on general information on the research	41/62	44/66	0.9919 (p = 0.9487)
**Man 2015**	optimised written patient information material vs. original patient information material	43/682	27/682	1.5926 (p = 0.0520)
**Hughes-Morley 2016**	Leaflet invitations sent to advertise PPIR vs. no leaflet invitations	216/5382	148/2800	Odds Ratio = 0.75^1^

Note

1. Hughes-Morley (2016) reported ORs and used a random effects logistic regression, which yielded OR = 0.75, 95% CI: 0.53 to 1.07, p = 0.013

#### 3.2.3 Non-randomised studies

Krusche *et al*. suggested that recruiting by adverts and posters showed no less efficacy than recruiting from GP referrals. In contrast, a study using electronic health records to remind GPs to approach potentially eligible patients was more efficient than recruitment by research staff in the clinic waiting room. The latter involved considerably more effort (more than 8000 patients were approached).[19] Le *et al*. also suggested that being contacted by clinical staff was more successful than being contacted by research staff. Among trials involving people with common mental disorders, GP referrals and contact by clinical staff were the most efficient and successful recruitment strategies and both resulted in an adequate number of patients for the size of a modern trial. Financial incentives are commonly used in commercially funded trials. The study done by DeBar suggested that neither different levels of financial incentives nor different lengths of assessment substantially affected recruitment rates. [23]

In an RCT of mindfulness versus treatment as usual (TAU) in women with depression, anxiety or stress, Woolhouse *et al*. used both more active (researcher approaching patients in clinic waiting room) and less active (invitations sent to potential participants) strategies.[15] The numbers of patients recruited were similar, despite 2,500 mailshots being sent compared with the researcher approaching 50 patients. A study comparing various forms of online recruitment for a preventive intervention for people with subthreshold depressive symptoms (as assessed by an online questionnaire) found that Google adverts recruited the highest number of participants (755 patients recruited). However, it was indicated that a total of 94,808 potential participants were approached, echoing findings by Krusche *et al*. suggesting that lower success rates may often be the case in recruitment via online advertisements. [16]

#### 3.2.4 Cost effectiveness of recruitment strategies

**Fig 2:**
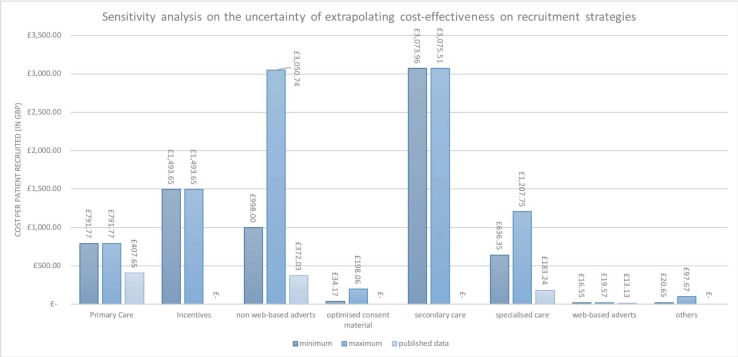
A sensitivity analysis on the uncertainty of extrapolating the cost-effectiveness on recruitment strategies.

The results of the cost-effectiveness of recruitment strategies are reported in Table 3. The strategy with the lowest cost per patient recruited was web-based advertisement (£13.41 per patient), followed by recruiting via specialised care (£183.24 per patient), non-web-based adverts (£372.03 per patient) and recruitment via primary care (£407.65 per patient). The sensitivity analysis considered the variation in cost according to the different strategies used. For instance, the cost of recruiting using health care providers depends on how much time is spent on recruitment. The two assumed levels of time commitment were part-time (3 hours/day) versus full-time (7.5 hours/day). Fig 2 shows the results of the costing and sensitivity analyses, in comparison with the cost-effectiveness reported with original data. As each study reported different information on costing the recruitment, even for similar strategies across different studies, costs obtained from available sources showed considerable variation. Shown below is an example of how recruitment cost was obtained, using a study by Morgan *et al*. (Table 4). Further details and sensitivity analysis are given in S1 Data.

**Table 3:** Summary of recruitment strategies across studies.

Recruitment strategy	Number of studies where strategy was used	Average cost per randomised participant (in GBP) with original data^1^	Number of times recruiting the most within study
**Web-based adverts^2^**	2	£13.41	2
**Via specialised care**	4	£183.24	1
**Via secondary care**	2	not reported	1
**Non-web-based adverts**	3	£372.03	1
**Financial incentives^3^**	1	not reported	1
**Via primary care**	4	£407.65	2
**Others**	4	not reported	0

Notes

1. The cost results account for the average exchange rates GBP/AUD and GBP/USD in year 2016, and inflation rates ofthe countries of publication from year of publication until 2016. (http://www.ukforex.co.uk/forex-tools/historical-rate-tools/yearly-average-rates; http://ination.stephenmorley.org/; U.S. Internal Revenue Service)

2. Results on web-based adverts included Morgan (2013), a study which used a number of different online resources to recruit patients.

3. Results on financial incentives included DeBar (2009), a study which used different incentives to recruit patients.

**Table 4:** An example of the detailed costing for a strategy used in Morgan 2013.

Study ID	Recruitment strategy	Description in original text	Resource used for costing	Calculation	Notes	Min. Cost (in GBP)	Max. Cost (in GBP)
**Morgan 2013**	links from webpage	"A new page of supporters was created to accommodate this requirement. This page thanked each organization or website that had helped promote the study to participants. Some websites were generous and included a link and blurb on their home page; others listed the website within a section of their site that contained links to other interesting websites."	Clicking on the webpage link, assumed cost zero.	assuming from 2hrs/day to full time responsible for mailing and posting, salary Band 7 £38,786. (£52/hr)	recruitment from Feb2010 to March 2011(13 months). However, no information on how many hours dedicated to such strategy.	25,740	38,786

### 3.3 Retention strategies

Table 5 summarises two studies identified that compared different strategies to improve postal response in surveys. On joining the trials, participants were randomised to followed up via different methods, and their response rates at follow-up were compared as a proxy for retention rates using the different methods (McLean 2014, Dirmaier 2007). McLean *et al*. investigated the effects of pre-notification (e.g. notifying participants in advance that they would be asked for information) and envelope ‘teaser’ (placement of a short message on the survey envelope) on increasing postal response rates in a bulimia nervosa mental health literacy survey. Dirmaier *et al*. conducted a randomised trial to find out whether small cash incentives and a shortened questionnaire helped increase postal response rates in a mailed follow-up survey one year after inpatient psychotherapeutic treatment for mental health patients. Both studies used a 2×2 factorial design to investigate the impact of strategies on postal response rates. Financial incentives, abridged questionnaire and pre-notification were suggested to be effective to increase postal response rates, but the effects were small.

**Table 5:** Summary of retention strategies.

Study ID	Retention strategy	Study period	Numbers approached	Numbers responded	Response rates	Cost information	Relative risk
**McLean 2014 [26]**	Prenotification (+), envelope teaser (-)	not reported	762	190	25%	$23.68/response	Marginal Prenotification RR = 1.165 (p = 0.027) Marginal Envelope Teaser RR = 0.955 (p = 0.508)
	Prenotification (+), envelope teaser (+)	not reported	747	167	22%	Not reported	
	Prenotification (-), envelope teaser (-)	not reported	750	150	20%	$26.25/response	
	Prenotification (-), envelope teaser (+)	not reported	747	154	21%	Not reported	
**Dirmaier 2007 [27]**	Financial incentive (+), abridged questionnaire (-)	1 year	832	458	55%	Not reported	Marginal Incentive RR = 1.146 (p < 0.0001) Marginal abridged questionnaire RR = 1.073 (p = 0.021)
	Financial incentive (+), abridged questionnaire (+)	1 year	845	500	59%	Not reported	
	Financial incentive (-), abridged questionnaire (-)	1 year	1045	502	48%	Not reported	
	Financial incentive (-), abridged questionnaire (+)	1 year	1103	569	52%	Not reported	

## 4. Discussion

The review identified only 3 eligible randomised comparative studies of alternative recruitment strategies in mental health clinical trials. None showed a statistically significant difference between using standard and optimised patient consent and information materials. Our findings were consistent with those of Treweek *et al*. The difference approached significance in one trial of recruitment using optimised patient information material compared with original patient material (Man 2015), although the effect was small. The 8 other studies included in the recruitment section of the review consisted of non-randomised, retrospective comparisons of different recruitment strategies. It is difficult to know whether the different strategies were employed in comparable ways in these studies, or for the same duration. Given the small number of randomised comparative studies identified, and the inconclusive results, this review suggests further research in this area may benefit trial recruitment. Two randomised studies comparing different retention strategies in mental health were identified (McLean 2014, Dirmaier 2007). Both involved different ways of maximising response rates to postal assessments. As follow-up assessment in RCTs is often carried out in the form of a questionnaire, the response rate to this type of assessment may be appropriate as a proxy for retention.

Prior to this review, we also piloted a search strategy that encompassed informed consent, recruitment, antipsychotics and randomised trials, attempting to review recruitment strategies in antipsychotic randomised trials. It generated approximately 2,000 records from MEDLINE, EMBASE, and CMR. However, after screening there was only 1 study (Jeste 2009) which met our criteria. Little attention has been paid to such methodological trials (e.g. using SWATs to increase the evidence base for trial decision-making) that endeavour to tackle some of the most common issues in mental health clinical trials.

The included studies showed substantial differences in strategies used, but also in clinical settings, mental health conditions and study design. We were not able to obtain a pooled estimate of recruitment efficacy of these strategies due to the non-randomised designs used, and the choice of analysis which could be used to assess the relative efficacy of different strategies was limited. It is therefore difficult to estimate the efficacy from beyond an individual level. Also, some included studies did not report numbers of potential participants approached by each strategy, e.g. the denominator for the efficiency measure of recruitment strategies (number recruited divided by number approached), and comparison between numerators should be made with caution, as some strategies have broader reach to the population and some studies required larger sample sizes. There were some interesting insights from the result of some recruitment studies, nevertheless. For instance, although clinical staff and GPs are often thought to be helpful in recruiting patients into randomised trials, here it was shown that they recruited no better than advertisements. The comparisons made were ad hoc, however, and in the absence of randomised controlled experiments, the area needs more rigorous investigation.

In this review, we also considered cost-effectiveness for each strategy based on numbers of participants recruited and cost incurred. It provided some useful information for public funded trials, which often work on a limited budget. However, it is worth noting that the choice of recruitment strategy should consider not solely cost-effectiveness, but also the study design, types of intervention and more importantly, population characteristics. For instance, we found that although using web-based advertisements showed merit in terms of efficacy and cost-effectiveness in recruitment, however the loss to follow-up in the population recruited via this method cannot be ignored. It is essential also to consider whether certain recruitment methods may identify a biased population. We also considered the uncertainty due to the inadequately reported cost information in the included studies, and performed sensitivity analysis of the costs obtained. The lack of a standard and transparent methodological framework for reporting the costs or resource use during recruitment has engendered considerable variations in the analysis and has led to challenges in interpreting the results. For instance, strategies that involved research assistants recruiting in clinic waiting rooms did not specify the total hours spent, therefore it was necessary to make assumptions regarding the numbers of hours spent per day on the recruitment task. Even for studies which employed similar recruitment strategies, reporting on resources used during recruitment varied tremendously, leading to considerable differences in costs obtained. Speich *et al*. also found in their systematic review that none of the included studies provided empirical resource use and cost data for all aspects of an RCT, and for trials that reported costs of recruitment, even similar recruitment strategies could cost different amounts across studies. Within a given category of recruitment strategies, for instance, the median cost of a mailed invitation was 228 USD, ranging from 15 to 1,116 USD per patient. [28]

Recently the ORRCA project (Online Resource for Recruitment research in Clinical triAls, www.orrca.org.uk) has attempted to bring together all the studies on recruitment into randomised trials by creating a searchable database. This initiative may help to inform trialists and recruiters of better ways to recruit patients into trials.[29] Also, Madurasinghe *et al*. provided guidelines for reporting embedded recruitment trials, for which a checklist based on the Consolidated Standards for Reporting Trials (CONSORT) statement 2010 was developed and several examples were listed. [30] Unlike the existing literature, this review has a focus on recruitment via different channels used as strategies described in the included studies, partly because of the inadequacy of the evidence available for mental health trials. It provides some evidence from a different perspective and makes suggestions regarding possible future research in this area. For instance, SWATs may be designed to compare the efficacy of recruitment by research staff with recruitment by clinical staff. Promoting the guidelines by Madurasinghe *et al*. will help to improve the quality of reporting for these methodological trials. Furthermore, it is also worth investigating the performance of different recruitment strategies with respect to other aspects of the trial, such as the population characteristics or adherence to the trial intervention, as these features also can determine a trial’s precision and efficiency. Some strategies may recruit a biased sample. For instance, using web-based adverts as a recruitment method in mental health trials may inadvertently recruit the “worried well” or those who do not sufficiently resemble real-world patients.

This study has the following limitations. Firstly, we only identified 3 randomised comparative studies of recruitment and two of retention. The rest compared different strategies without randomisation and this may diminish the internal validity of their findings. Secondly, out of 13 identified studies, the majority were in depression-related illnesses. The limited number of studies involving people with diagnoses of severe mental illnesses such as bipolar disorders or schizophrenia, reduces the generalisability of the review. It highlights the need for more research in this area, since there are many challenges to recruitment within this group of people. Moreover, there were no eligible RCTs aimed at improving retention within randomised controlled trials. We included 2 studies which focus on improving postal response rates in follow-up, despite the fact they were not set within a randomised clinical trial. However, since they used a randomised design to assess methods to enhance response rates, we believe they contribute useful information, although clearly more studies are needed to address retention issues in randomised trials and in studies that use face to face assessments rather than postal questionnaires. Lastly, lack of reported information on costs in many of the included studies means there is considerable uncertainty in our findings on cost-effectiveness.

## 5. Conclusion

This review discusses the different strategies to improve recruitment and retention in mental health clinical trials. The recruitment studies included showed substantial variation in strategies, clinical settings, mental health conditions and study design. It is difficult to assess the overall efficacy of any particular recruitment strategy as some strategies that worked well for a particular population may not work as well for others. Paying attention to the accessibility of information and consent materials (optimisation) may help improve recruitment. Recruitment by clinical staff and non-web-based adverts showed some efficiency and success in certain circumstances. Pre-notification, abridged questionnaires and financial incentives have small positive effects on retention rates in postal surveys. The limited number of eligible studies identified suggests that more research in this area is needed given its important implications.

## Supporting information

S1 Checklist PRISMA checklist. (DOC)

S1 File Protocol and search strategies. (DOCX)

S2 File Risk of bias assessment. (DOCX)

S1 Supplementary Description of the included studies. (DOCX)

S1 Data Data extracted from the included studies. (XLSX)
