# Supplementary material for: Recruitment and retention strategies in mental health trials – A systematic review
Source: PLoS One. 2018 Aug 29;13(8):e0203127. doi: 10.1371/journal.pone.0203127 (PMC6114918; doi:10.1371/journal.pone.0203127)
Supplement: S1 File — (DOCX) [file pone.0203127.s002.docx]

# Objectives

To investigate and evaluate different approaches to recruiting and retaining patients into mental health randomised clinical trials (RCT) and what may be done to improve recruitment and retention rate in RCT of such kinds. In addition, to discuss how different these strategies used in RCTs for mental illness enhance recruitment and retention rate.

# Background

Recruitment in randomised clinical trials is challenging and delayed recruitment can give rise to a series of issues, such as reducing the power of the study and additional costs and extension of study period. Inadequate or ineffective recruitment may often result in early-termination of a trial and the failure in answering an important clinical question. As the gold standard of clinical research, randomised clinical trials often fail in recruiting enough subjects, particularly in mental health area. Lost to follow-up and patients dropouts can also cause a reduced study power in discovering the difference between trial arms. Although an increasing amount of research has contributed to dealing with missing data in clinical trials using various statistical techniques, the risk of bias due to missing data still cannot be avoided by such imputations. It is suggested that a less than 5% loss to follow-up may lead to minimum bias, while 20% lost to follow-up can pose a threat to a trial's validity. Some modern trials aim to reduce this risk by increasing the sample size by 20%, which in return increases the recruitment difficulties.

This review hopes to contribute to improving both the recruitment and retention rate for mental illness clinical trials.

# Searching

MEDLINE, EMBASE, PsycINFO and Cochrane Methodology Register(CMR) are used for search of articles published in English language since inception of each database. The search strategy used in MEDLINE includes MeSH terms such as *exp "bipolar and related disorders"/ or "schizophrenia spectrum and other psychotic disorders"/ or exp schizophrenia/,* other search keywords include (randomi* adj2 clinical trial*).ti.. Full search strategy is included in the appendix.

# Study Selection

Randomised controlled trials of interventions to improve recruitment and retention for mental illness randomised controlled trials.

## Inclusion

- Comparative studies in which at least two different recruitment or retention strategies were compared in an RCT are eligible for inclusion.
- RCTs with adults are eligible for inclusion.
- Mental health illness is defined as conditions that are catogrised in DSM-5 or ICD-10 criteria.
- Studies are funded by public healthcare sector as well as ones by industry are eligible for inclusion.
- Studies to improve recruitment to cluster randomised trials, pilot stage of trials and feasibility studies need further screening for inclusion.
- Strategies both in real settings and in hypothetical settings (studies that ask potential patients’ consent to participate in a trial if it was run but without the trial actually running) are eligible.
- As for specific recruitment strategies, we include all strategies that aim to improve recruitment of participants, mainly below but not limited to:
- Newsletters (including mailshots)
- Regular visits/telephone calls
- Posters in clinics/wards
- Amendments on protocol
- Presentations to appropriate groups
- Presentations at conferences
- Extra staffing
- Investigators’ meeting/recruiting staff meetings
- Trial material customized to specific sites
- Investigators’ visits to centers
- Letter to individuals
- Advertisement in newspapers
- Resource manual for recruiters
- Training
- Monetary incentives

## Exclusion

- Studies on non-adults (i.e. children) are excluded.
- Studies that did not randomise patients into different groups are excluded.
- Studies in which mental illness as co-occurrence with primary conditions (i.e. cardiovascular diseases) are excluded.
- Studies which did not report comparison on interventions to improve recruitment nor retention for RCTs are excluded.
- Studies that focused on people with substance misuse problems where comorbid mental health conditions had not been assessed using DSM or ICD-10 criteria are excluded.

# Outcome measures

## Primary outcomes

For recruitment trials: Difference in numbers of patients recruited using each strategy

For retention trials: numbers of patients who were included in the final analysis over the numbers of patients who were randomised to each retention arm.

## Secondary outcomes

- Cost effectiveness of different strategies
- Difficulties during recruitment
- Benefits using different strategies

Analysis will be described in a separate statistical analysis plan.

# Assessment of study quality

For each included study, their adequacy of allocation concealment will be assessed (adequate, unclear and inadequate). Completeness of result reporting, loss to follow-up, risk of bias and other aspects will also be assessed. Completeness of reporting will be assessed by the quality of information on participants, intervention, comparator and outcome, as well as whether allocation was concealed. A CONSORT diagram will be used to record the flow of participants through the trial.

# Data extraction

A data extraction form will be created to collect the information for the outcomes in each eligible study. Data included in the “outcome measures” section will be collected. Authors of the studies will also be contacted for necessary but unpublished information.

# Appendix

## Search Strategies

### MEDLINE(Ovid)

1 Patient Selection/ (55467)

2 ((participat$ or recruit$ or enrol$) adj4 trial?).tw. (20036)

3 Informed Consent/ (33169)

4 informed consent.tw. (28318)

5 recruitment.ab. /freq=2 (21355)

6 participation.ab. /freq=2 (20980)

7 (minimi$ adj2 attrition).ab,ti. (85)

8 (prevent$ adj2 attrition).ab,ti. (89)

9 (lessen$ adj2 attrition).ab,ti. (2)

10 (decreas$ adj2 attrition).ab,ti. (97)

11 (reduc$ adj2 attrition).ab,ti. (387)

12 (minimi$ adj2 drop-out).ab,ti. (7)

13 (prevent$ adj2 drop-out).ab,ti. (24)

14 (lessen$ adj2 drop-out).ab,ti. (1)

15 (decreas$ adj2 drop-out).ab,ti. (17)

16 (reduc$ adj2 drop-out).ab,ti. (66)

17 (minimi$ adj2 drop-out$).ab,ti. (9)

18 (prevent$ adj2 drop-out$).ab,ti. (26)

19 (lessen$ adj2 drop-out$).ab,ti. (1)

20 (decreas$ adj2 drop-out$).ab,ti. (22)

21 (reduc$ adj2 drop-out$).ab,ti. (82)

22 (minimi$ adj2 drop$-out).ab,ti. (9)

23 (prevent$ adj2 drop$-out).ab,ti. (28)

24 (lessen$ adj2 drop$-out).ab,ti. (1)

25 (decreas$ adj2 drop$-out).ab,ti. (20)

26 (reduc$ adj2 drop$-out).ab,ti. (72)

27 (minimi$ adj2 dropout$).ab,ti. (33)

28 (prevent$ adj2 dropout$).ab,ti. (111)

29 (lessen$ adj2 dropout$).ab,ti. (0)

30 (decreas$ adj2 dropout$).ab,ti. (63)

31 (reduc$ adj2 dropout$).ab,ti. (203)

32 (strateg$ adj2 drop$-out).ab,ti. (2)

33 (strateg$ adj2 dropout$).ab,ti. (8)

34 (loss adj2 follow-up).ab,ti. (3183)

35 (lost adj2 follow-up).ab,ti. (14548)

36 (loss adj2 followup).ab,ti. (48)

37 (lost adj2 followup).ab,ti. (652)

38 (minimi$ adj2 withdrawal).ab,ti. (93)

39 (prevent$ adj2 withdrawal).ab,ti. (404)

40 (lessen$ adj2 withdrawal).ab,ti. (9)

41 (decreas$ adj2 withdrawal).ab,ti. (687)

42 (reduc$ adj2 withdrawal).ab,ti. (1092)

43 (minimi$ adj2 withdrawal$).ab,ti. (97)

44 (prevent$ adj2 withdrawal$).ab,ti. (412)

45 (lessen$ adj2 withdrawal$).ab,ti. (9)

46 (decreas$ adj2 withdrawal$).ab,ti. (693)

47 (reduc$ adj2 withdrawal$).ab,ti. (1114)

48 (strateg$ adj2 attrition).ab,ti. (17)

49 (strateg$ adj2 drop-out).ab,ti. (1)

50 (strateg$ adj2 dropout).ab,ti. (5)

51 (strateg$ adj2 follow-up).ab,ti. (1092)

52 (strateg$ adj2 followup).ab,ti. (22)

53 (increas$ adj2 retention).ab,ti. (4080)

54 (encourag$ adj2 retention).ab,ti. (77)

55 (maximi$ adj2 retention).ab,ti. (152)

56 (promot$ adj2 retention).ab,ti. (727)

57 (improv$ adj2 retention).ab,ti. (2637)

58 (strateg$ adj2 response$).ab,ti. (2016)

59 (strateg$ adj2 (questionnaire$ adj3 response$)).ab,ti. (9)

60 (increas$ adj2 (questionnaire$ adj3 response$)).ab,ti. (40)

61 (encourag$ adj2 (questionnaire$ adj3 response$)).ab,ti. (1)

62 (maximi$ adj2 (questionnaire$ adj3 response$)).ab,ti. (3)

63 (promot$ adj2 (questionnaire$ adj3 response$)).ab,ti. (1)

64 (improv$ adj2 (questionnaire$ adj3 response$)).ab,ti. (23)

65 (increas$ adj2 response$).ab,ti. (36535)

66 (encourag$ adj2 response$).ab,ti. (601)

67 (maximi$ adj2 response$).ab,ti. (530)

68 (promot$ adj2 response$).ab,ti. (3924)

69 (improv$ adj2 response$).ab,ti. (11236)

70 (retention adj2 strateg$).ab,ti. (692)

71 retention rate$.ab,ti. (3246)

72 (retention adj2 method$).ab,ti. (412)

73 (retention adj2 technique$).ab,ti. (177)

74 attrition rate$.ab,ti. (1748)

75 (questionnaire$ adj3 (response$ adj2 method$)).ab,ti. (29)

76 (questionnaire$ adj3 (response adj2 technique$)).ab,ti. (3)

77 (questionnaire adj response rate$).ab,ti. (689)

78 (difficult$ adj2 (retain$ or retention)).ab,ti. (245)

79 Patient Dropouts/ (7244)

80 1 or 2 or 3 or 4 or 5 or 6 or 7 or 8 or 9 or 10 or 11 or 12 or 13 or 14 or 15 or 16 or 17 or 18 or 19 or 20 or 21 or 22 or 23 or 24 or 25 or 26 or 27 or 28 or 29 or 30 or 31 or 32 or 33 or 34 or 35 or 36 or 37 or 38 or 39 or 40 or 41 or 42 or 43 or 44 or 45 or 46 or 47 or 48 or 49 or 50 or 51 or 52 or 53 or 54 or 55 or 56 or 57 or 58 or 59 or 60 or 61 or 62 or 63 or 64 or 65 or 66 or 67 or 68 or 69 or 70 or 71 or 72 or 73 or 74 or 75 or 76 or 77 or 78 or 79 (256317)

81 randomized controlled trial.pt. (425254)

82 controlled clinical trial.pt. (91288)

83 randomized.ab. (364059)

84 placebo.ab. (176659)

85 clinical trials as topic.sh. (178334)

86 randomly.ab. (260090)

87 trial.ti. (158896)

88 81 or 82 or 83 or 84 or 85 or 86 or 87 (1054276)

89 exp animals/ not humans.sh. (4280763)

90 88 not 89 (972053)

91 80 and 90 (43387)

92 exp "Feeding and Eating Disorders"/ (25424)

93 exp Anorexia nervosa/ (11498)

94 exp Bulimia Nervosa/ (1865)

95 exp Suicide, attempted/ (16837)

96 exp Self mutilation/ (3121)

97 exp Self-injurious behavior/ (59536)

98 exp Mood disorders/ (103490)

99 exp Bipolar disorder/ (34994)

100 exp Neurotic disorders/ (17974)

101 exp Depressive disorder/ (92517)

102 exp Dysthymic disorder/ (1062)

103 exp depression/ (90326)

104 exp Seasonal affective disorder/ (1135)

105 exp anxiety/ or exp anxiety disorders/ or exp anxiety, separation/ or exp dental anxiety/ (128930)

106 exp panic/ or exp panic disorder/ (8717)

107 exp phobic disorders/ (10008)

108 exp combat disorders/ or exp stress disorders, post-traumatic/ (26209)

109 exp Somatoform disorders/ (17354)

110 exp Hypochondriasis/ (2197)

111 exp Hysteria/ (3526)

112 exp Conversion disorder/ (2612)

113 exp munchausen syndrome/ or munchausen syndrome by proxy/ (1739)

114 exp Neurasthenia/ (1346)

115 exp Fatigue syndrome, chronic/ (4796)

116 exp Obsessive-compulsive disorder/ (12603)

117 exp Obsessive behavior/ (1195)

118 exp Compulsive behavior/ (9361)

119 exp Stress, psychological/ (104897)

120 *Mental Disorders/ (111864)

121 exp schizophrenia/ (98181)

122 exp paranoid disorders/ (3903)

123 schizo$.mp. (153123)

124 hebephreni$.mp. (274)

125 oligophreni$.mp. (1102)

126 psychotic$.mp. (60934)

127 psychos#s.mp. (46522)

128 (chronic$ adj mental$).ti,ab. (1841)

129 (sever$ adj mental).ti,ab. (7050)

130 (mental$ adj disorder$).ti,ab. (29226)

131 (mental$ adj ill$).ti,ab. (28537)

132 (emotion$ adj disorder$).ti,ab. (2168)

133 exp "schizophrenia spectrum and other psychotic disorders"/ (137268)

134 or/92-133 (759610)

135 91 and 134 (3448)

136 exp dissociative disorders/ (3797)

137 exp personality disorders/ (37882)

138 134 or 136 or 137 (777550)

139 91 and 138 (3490)

### EMBASE(Ovid)

1 ((participat$ or recruit$ or enrol$ or enter$ or entry) and (trial? or study)).ti. (13443)

2 (select$ adj3 (participants or patients or controls)).tw. (142299)

3 recruit$.ab. /freq=2 (60587)

4 participat$.ab. /freq=2 (73083)

5 research.tw. (1342128)

6 2 and (3 or 4 or 5) (9664)

7 (informed consent or consent process$ or consent procedure?).tw. (53284)

8 1 or 6 or 7 (75736)

9 (minimi$ adj2 attrition).ab,ti. (91)

10 (prevent$ adj2 attrition).ab,ti. (101)

11 (lessen$ adj2 attrition).ab,ti. (2)

12 (decreas$ adj2 attrition).ab,ti. (101)

13 (reduc$ adj2 attrition).ab,ti. (480)

14 (minimi$ adj2 drop-out).ab,ti. (9)

15 (prevent$ adj2 drop-out).ab,ti. (39)

16 (lessen$ adj2 drop-out).ab,ti. (1)

17 (decreas$ adj2 drop-out).ab,ti. (31)

18 (reduc$ adj2 drop-out).ab,ti. (101)

19 (minimi$ adj2 drop-out$).ab,ti. (13)

20 (prevent$ adj2 drop-out$).ab,ti. (47)

21 (lessen$ adj2 drop-out$).ab,ti. (1)

22 (decreas$ adj2 drop-out$).ab,ti. (40)

23 (reduc$ adj2 drop-out$).ab,ti. (122)

24 (minimi$ adj2 drop$-out).ab,ti. (10)

25 (prevent$ adj2 drop$-out).ab,ti. (46)

26 (lessen$ adj2 drop$-out).ab,ti. (1)

27 (decreas$ adj2 drop$-out).ab,ti. (36)

28 (reduc$ adj2 drop$-out).ab,ti. (112)

29 (minimi$ adj2 dropout$).ab,ti. (40)

30 (prevent$ adj2 dropout$).ab,ti. (128)

31 (lessen$ adj2 dropout$).ab,ti. (1)

32 (decreas$ adj2 dropout$).ab,ti. (77)

33 (reduc$ adj2 dropout$).ab,ti. (237)

34 (strateg$ adj2 drop$-out).ab,ti. (4)

35 (strateg$ adj2 dropout$).ab,ti. (11)

36 (loss adj2 follow-up).ab,ti. (4737)

37 (lost adj2 follow-up).ab,ti. (22872)

38 (loss adj2 followup).ab,ti. (98)

39 (lost adj2 followup).ab,ti. (891)

40 (minimi$ adj2 withdrawal).ab,ti. (144)

41 (prevent$ adj2 withdrawal).ab,ti. (504)

42 (lessen$ adj2 withdrawal).ab,ti. (9)

43 (decreas$ adj2 withdrawal).ab,ti. (868)

44 (reduc$ adj2 withdrawal).ab,ti. (1416)

45 (minimi$ adj2 withdrawal$).ab,ti. (151)

46 (prevent$ adj2 withdrawal$).ab,ti. (514)

47 (lessen$ adj2 withdrawal$).ab,ti. (9)

48 (decreas$ adj2 withdrawal$).ab,ti. (878)

49 (reduc$ adj2 withdrawal$).ab,ti. (1443)

50 (strateg$ adj2 attrition).ab,ti. (17)

51 (strateg$ adj2 drop-out).ab,ti. (3)

52 (strateg$ adj2 dropout).ab,ti. (9)

53 (strateg$ adj2 follow-up).ab,ti. (1539)

54 (strateg$ adj2 followup).ab,ti. (31)

55 (increas$ adj2 retention).ab,ti. (4810)

56 (encourag$ adj2 retention).ab,ti. (84)

57 (maximi$ adj2 retention).ab,ti. (169)

58 (promot$ adj2 retention).ab,ti. (854)

59 (improv$ adj2 retention).ab,ti. (2932)

60 (strateg$ adj2 response$).ab,ti. (2259)

61 (strateg$ adj2 (questionnaire$ adj3 response$)).ab,ti. (10)

62 (increas$ adj2 (questionnaire$ adj3

response$)).ab,ti. (53)

63 (encourag$ adj2 (questionnaire$ adj3 response$)).ab,ti. (1)

64 (maximi$ adj2 (questionnaire$ adj3 response$)).ab,ti. (5)

65 (promot$ adj2 (questionnaire$ adj3 response$)).ab,ti. (1)

66 (improv$ adj2 (questionnaire$ adj3 response$)).ab,ti. (30)

67 (increas$ adj2 response$).ab,ti. (42610)

68 (encourag$ adj2 response$).ab,ti. (839)

69 (maximi$ adj2 response$).ab,ti. (709)

70 (promot$ adj2 response$).ab,ti. (4797)

71 (improv$ adj2 response$).ab,ti. (15843)

72 (retention adj2 strateg$).ab,ti. (723)

73 retention rate$.ab,ti. (4255)

74 (retention adj2 method$).ab,ti. (874)

75 (retention adj2 technique$).ab,ti. (198)

76 attrition rate$.ab,ti. (2200)

77 (questionnaire$ adj3 (response$ adj2 method$)).ab,ti. (62)

78 (questionnaire$ adj3 (response adj2 technique$)).ab,ti. (2)

79 (questionnaire adj response rate$).ab,ti. (950)

80 (difficult$ adj2 (retain$ or retention)).ab,ti. (324)

81 Participant Dropouts/ (0)

82 9 or 10 or 11 or 12 or 13 or 14 or 15 or 16 or 17 or 18 or 19 or 20 or 21 or 22 or 23 or 24 or 25 or 26 or 27 or 28 or 29 or 30 or 31 or 32 or 33 or 34 or 35 or 36 or 37 or 38 or 39 or 40 or 41 or 42 or 43 or 44 or 45 or 46 or 47 or 48 or 49 or 50 or 51 or 52 or 53 or 54 or 55 or 56 or 57 or 58 or 59 or 60 or 61 or 62 or 63 or 64 or 65 or 66 or 67 or 68 or 69 or 70 or 71 or 72 or 73 or 74 or 75 or 76 or 77 or 78 or 79 or 80 or 81 (116847)

83 8 or 82 (191589)

84 random$.tw. (1094465)

85 placebo$.ti,ab,sh. (365359)

86 double-blind$.tw. (164070)

87 84 or 85 or 86 (1311578)

88 eating disorder/ or anorexia nervosa/ or binge eating disorder/ or bulimia/ (39306)

89 exp suicidal behavior/ (78654)

90 automutilation/ (12559)

91 exp mood disorder/ (396029)

92 exp neurosis/ (52551)

93 exp anxiety disorder/ (176035)

94 anxiety/ (145205)

95 dental anxiety/ (2057)

96 exp psychosomatic disorder/ (37699)

97 chronic fatigue syndrome/ (8104)

98 chronic stress/ or emotional stress/ or mental stress/ (85748)

99 *mental disease/ (93464)

100 exp psychosis/ (239967)

101 schizo$.mp. (195580)

102 hebephreni$.mp. (776)

103 oligophreni$.mp. (1526)

104 psychotic$.mp. (43108)

105 psychos#s.mp. (110772)

106 (chronic$ adj mental$).ti,ab. (2162)

107 (sever$ adj mental).ti,ab. (8886)

108 (mental$ adj disorder$).ti,ab. (36208)

109 (mental$ adj ill$).ti,ab. (34176)

110 (emotion$ adj disorders$).ti,ab. (2345)

111 or/88-110 (1051509)

112 83 and 87 and 111 (2861)

### PsycINFO(Ovid)

1 Patient Selection/ (186)

2 ((participat$ or recruit$ or enrol$) adj4 trial?).tw. (3138)

3 1 or 2 (3320)

4 Informed Consent/ (3704)

5 informed consent.tw. (6695)

6 4 or 5 (7730)

7 exp Clinical Trial/ (9713)

8 Experimental Subjects/ (3778)

9 (trial? or study or studies or research).tw. (2039814)

10 7 or 8 or 9 (2040228)

11 3 or (6 and 10) (8130)

12 (minimi$ adj2 attrition).ab,ti. (49)

13 (prevent$ adj2 attrition).ab,ti. (66)

14 (lessen$ adj2 attrition).ab,ti. (2)

15 (decreas$ adj2 attrition).ab,ti. (95)

16 (reduc$ adj2 attrition).ab,ti. (271)

17 (minimi$ adj2 drop-out).ab,ti. (5)

18 (prevent$ adj2 drop-out).ab,ti. (52)

19 (lessen$ adj2 drop-out).ab,ti. (0)

20 (decreas$ adj2 drop-out).ab,ti. (16)

21 (reduc$ adj2 drop-out).ab,ti. (57)

22 (minimi$ adj2 drop-out$).ab,ti. (5)

23 (prevent$ adj2 drop-out$).ab,ti. (55)

24 (lessen$ adj2 drop-out$).ab,ti. (0)

25 (decreas$ adj2 drop-out$).ab,ti. (19)

26 (reduc$ adj2 drop-out$).ab,ti. (67)

27 (minimi$ adj2 drop$-out).ab,ti. (5)

28 (prevent$ adj2 drop$-out).ab,ti. (65)

29 (lessen$ adj2 drop$-out).ab,ti. (0)

30 (decreas$ adj2 drop$-out).ab,ti. (20)

31 (reduc$ adj2 drop$-out).ab,ti. (62)

32 (minimi$ adj2 dropout$).ab,ti. (15)

33 (prevent$ adj2 dropout$).ab,ti. (317)

34 (lessen$ adj2 dropout$).ab,ti. (0)

35 (decreas$ adj2 dropout$).ab,ti. (99)

36 (reduc$ adj2 dropout$).ab,ti. (226)

37 (strateg$ adj2 drop$-out).ab,ti. (6)

38 (strateg$ adj2 dropout$).ab,ti. (27)

39 (loss adj2 follow-up).ab,ti. (355)

40 (lost adj2 follow-up).ab,ti. (597)

41 (loss adj2 followup).ab,ti. (8)

42 (lost adj2 followup).ab,ti. (7)

43 (minimi$ adj2 withdrawal).ab,ti. (18)

44 (prevent$ adj2 withdrawal).ab,ti. (139)

45 (lessen$ adj2 withdrawal).ab,ti. (9)

46 (decreas$ adj2 withdrawal).ab,ti. (298)

47 (reduc$ adj2 withdrawal).ab,ti. (457)

48 (minimi$ adj2 withdrawal$).ab,ti. (19)

49 (prevent$ adj2 withdrawal$).ab,ti. (144)

50 (lessen$ adj2 withdrawal$).ab,ti. (9)

51 (decreas$ adj2 withdrawal$).ab,ti. (303)

52 (reduc$ adj2 withdrawal$).ab,ti. (463)

53 (strateg$ adj2 attrition).ab,ti. (20)

54 (strateg$ adj2 drop-out).ab,ti. (3)

55 (strateg$ adj2 dropout).ab,ti. (26)

56 (strateg$ adj2 follow-up).ab,ti. (127)

57 (strateg$ adj2 followup).ab,ti. (2)

58 (increas$ adj2 retention).ab,ti. (1089)

59 (encourag$ adj2 retention).ab,ti. (57)

60 (maximi$ adj2 retention).ab,ti. (64)

61 (promot$ adj2 retention).ab,ti. (343)

62 (improv$ adj2 retention).ab,ti. (1261)

63 (strateg$ adj2 response$).ab,ti. (2034)

64 (strateg$ adj2 (questionnaire$ adj3

response$)).ab,ti. (7)

65 (increas$ adj2 (questionnaire$ adj3 response$)).ab,ti. (16)

66 (encourag$ adj2 (questionnaire$ adj3 response$)).ab,ti. (1)

67 (maximi$ adj2 (questionnaire$ adj3 response$)).ab,ti. (4)

68 (promot$ adj2 (questionnaire$ adj3 response$)).ab,ti. (0)

69 (improv$ adj2 (questionnaire$ adj3 response$)).ab,ti. (12)

70 (increas$ adj2 response$).ab,ti. (7838)

71 (encourag$ adj2 response$).ab,ti. (169)

72 (maximi$ adj2 response$).ab,ti. (140)

73 (promot$ adj2 response$).ab,ti. (365)

74 (improv$ adj2 response$).ab,ti. (1542)

75 (retention adj2 strateg$).ab,ti. (513)

76 retention rate$.ab,ti. (1674)

77 (retention adj2 method$).ab,ti. (172)

78 (retention adj2 technique$).ab,ti. (16)

79 attrition rate$.ab,ti. (1192)

80 (questionnaire$ adj3 (response$ adj2 method$)).ab,ti. (20)

81 (questionnaire$ adj3 (response adj2 technique$)).ab,ti. (3)

82 (questionnaire adj response rate$).ab,ti. (175)

83 (difficult$ adj2 (retain$ or retention)).ab,ti. (147)

84 exp Treatment Dropouts/ (2227)

85 12 or 13 or 14 or 15 or 16 or 17 or 18 or 19 or 20 or 21 or 22 or 23 or 24 or 25 or 26 or 27 or 28 or 29 or 30 or 31 or 32 or 33 or 34 or 35 or 36 or 37 or 38 or 39 or 40 or 41 or 42 or 43 or 44 or 45 or 46 or 47 or 48 or 49 or 50 or 51 or 52 or 53 or 54 or 55 or 56 or 57 or 58 or 59 or 60 or 61 or 62 or 63 or 64 or 65 or 66 or 67 or 68 or 69 or 70 or 71 or 72 or 73 or 74 or 75 or 76 or 77 or 78 or 79 or 80 or 81 or 82 or 83 or 84 (22941)

86 double-blind.ab,ti. (19918)

87 "random$ assigned.".ab,ti. (28515)

88 control.ab,ti. (356849)

89 86 or 87 or 88 (389728)

90 exp Experimental Attrition/ (365)

91 85 or 90 (23177)

92 89 and 91 (3791)

93 exp eating disorders/ (25722)

94 exp attempted suicide/ (8675)

95 self-mutilation/ (1105)

96 exp Self-Injurious Behavior/ (4198)

97 exp affective disorders/ (136500)

98 exp neurosis/ (7571)

99 anxiety disorders/ (15529)

100 exp somatoform disorders/ (11519)

101 exp HYSTERIA/ (1981)

102 munchausen syndrome/ or munchausen syndrome by proxy/ (321)

103 exp chronic fatigue syndrome/ (1697)

104 exp psychological stress/ (8067)

105 *mental disorders/ (58903)

106 exp psychosis/ (100398)

107 schizoaffective disorder/ (2760)

108 schizo$.mp. (119741)

109 hebephreni$.mp. (533)

110 oligophreni$.mp. (520)

111 psychotic$.mp. (41118)

112 psychos#s.mp. (54151)

113 (chronic$ adj mental$).ti,ab. (2443)

114 (severe adj mental).ti,ab. (6322)

115 (mental$ adj disorder$).ti,ab. (43030)

116 (mental$ adj ill$).ti,ab. (40799)

117 (emotion$ adj disorder$).ti,ab. (3073)

118 or/93-117 (429478)

119 92 and 118 (492)

120 exp dissociative disorders/ (4873)

121 exp Personality Disorders/ (30149)

122 118 or 120 or 121 (452579)

123 122 and 92 (508)

### Cochrane Methodology Register(CMR)

#1 (minimi* near/2 attrition):ab,ti

#2 (prevent* near/2 attrition):ab,ti

#3 (lessen* near/2 attrition):ab,ti

#4 (decreas* near/2 attrition):ab,ti

#5 (reduc* near/2 attrition):ab,ti

#6 (minimi* near/2 drop-out):ab,ti

#7 (prevent* near/2 drop-out):ab,ti

#8 (lessen* near/2 drop-out):ab,ti

#9 (decreas* near/2 drop-out):ab,ti

#10 (reduc* near/2 drop-out):ab,ti

#11 (minimi* near/2 drop-out*):ab,ti

#12 (prevent* near/2 drop-out*):ab,ti

#13 (lessen* near/2 drop-out*):ab,ti

#14 (decreas* near/2 drop-out*):ab,ti

#15 (reduc* near/2 drop-out*):ab,ti

#16 (minimi* near/2 drop*-out):ab,ti

#17 (prevent* near/2 drop*-out):ab,ti

#18 (lessen* near/2 drop*-out):ab,ti

#19 (decreas* near/2 drop*-out):ab,ti

#20 (reduc* near/2 drop*-out):ab,ti

#21 (minimi* near/2 dropout*):ab,ti

#22 (prevent* near/2 dropout*):ab,ti

#23 (lessen* near/2 dropout*):ab,ti

#24 (decreas* near/2 dropout*):ab,ti

#25 (reduc* near/2 dropout*) .ab,ti

#26 (strateg* near/2 (questionnaire* near/3 response*)):ab,ti

#27 (increas* near/2 (questionnaire* near/3 response*)):ab,ti

#28 (encourag* near/2 (questionnaire* near/3 response*)):ab,ti

#29 (maximi* near/2 (questionnaire* near/3 response*)):ab,ti

#30 (promot* near/2 (questionnaire* near/3 response*)):ab,ti

#31 (improv* near/2 (questionnaire* near/3 response*)):ab,ti

#32 (increas* near/2 response*):ab,ti

#33 (encourag* near/2 response*):ab,ti

#34 (maximi* near/2 response*):ab,ti

#35 (promot* near/2 response*):ab,ti

#36 (improv* near/2 response*):ab,ti

#37 (retention near/2 strateg*):ab,ti

#38 retention rate*:ab,ti

#39 (retention near/2 method*):ab,ti

#40 (retention near/2 technique*):ab,ti

#41 attrition rate*:ab,ti

#42 (questionnaire* near/3 (response* near/2 method*)):ab,ti

#43 (questionnaire* near/3 (response near/2 technique*)):ab,ti

#44 (questionnaire near response rate*):ab,ti (1145)

#45 (difficult* near/2 (retain* or retention)):ab,ti

#46 MeSH descriptor: [Patient Dropouts] explode all trees

#47 (strateg* near/2 drop*-out):ab,ti

#48 (strateg* near/2 dropout*):ab,ti

#49 (loss near/2 follow-up):ab,ti

#50 (lost near/2 follow-up):ab,ti

#51 (loss near/2 followup):ab,ti

#52 (lost near/2 followup):ab,ti

#53 (minimi* near/2 withdrawal):ab,ti

#54 (prevent* near/2 withdrawal):ab,ti

#55 (lessen* near/2 withdrawal):ab,ti

#56 (decreas* near/2 withdrawal):ab,ti

#57 (reduc* near/2 withdrawal):ab,ti

#58 (minimi* near/2 withdrawal*):ab,ti

#59 (prevent* near/2 withdrawal*):ab,ti

#60 (lessen* near/2 withdrawal*):ab,ti

#61 (decreas* near/2 withdrawal*):ab,ti

#62 (reduc* near/2 withdrawal*):ab,ti

#63 (strateg* near/2 attrition):ab,ti

#64 (strateg* near/2 drop-out):ab,ti

#65 (strateg* near/2 dropout):ab,ti

#66 (strateg* near/2 follow-up):ab,ti

#67 (strateg* near/2 followup):ab,ti

#68 (increas* near/2 retention):ab,ti

#69 (encourag* near/2 retention):ab,ti

#70 (maximi* near/2 retention):ab,ti

#71 (promot* near/2 retention):ab,ti

#72 (improv* near/2 retention):ab,ti

#73 (strateg* near/2 response*):ab,ti

#74 "accrual and sample size":kw or "attitudes to trials":kw or "informed consent":kw

#75 (participat* or recruit* or enrol* or select*) near/8 (trial* or research or study):ti or (participat* or recruit* or enrol* or select*) near/8 (trial* or research or study):ab

#76 #1 or #2 or #3 or #4 or #5 or #6 or #7 or #8 or #9 or #10 or #11 or #12 or #13 or #14 or #15 or #16 or #17 or #18 or #19 or #20 or #21 or #22 or #23 or #24 or #25 or #26 or #27 or #28 or #29 or #30 or #31 or #32 or #33 or #34 or #35 or #36 or #37 or #38 or #39 or #40 or #41 or #42 or #43 or #44 or #45 or #46 or #47 or #48 or #49 or #50 or #51 or #52 or #53 or #54 or #55 or #56 or #57 or #58 or #59 or #60 or #61 or #62 or #63 or #64 or #65 or #66 or #67 or #68 or #69 or #70 or #71 or #72 or #73 or #74 or #75

#77 "accrual and sample size":kw or "attitudes to trials":kw or "informed consent":kw

#78 (participat* or recruit* or enrol* or select*) near/8 (trial* or research or study):ti or (participat* or recruit* or enrol* or select*) near/8 (trial* or research or study):ab

#79 (#77 or #78 or #76)

#80 MeSH descriptor: [Anorexia Nervosa] explode all trees

#81 MeSH descriptor: [Feeding and Eating Disorders] explode all trees

#82 MeSH descriptor: [Bulimia Nervosa] explode all trees

#83 MeSH descriptor: [Suicide, Attempted] explode all trees

#84 MeSH descriptor: [Self Mutilation] explode all trees

#85 MeSH descriptor: [Self-Injurious Behavior] explode all trees

#86 MeSH descriptor: [Mood Disorders] explode all trees

#87 MeSH descriptor: [Bipolar Disorder] explode all trees

#88 MeSH descriptor: [Neurotic Disorders] explode all trees

#89 MeSH descriptor: [Depressive Disorder] explode all trees

#90 MeSH descriptor: [Depression] explode all trees

#91 MeSH descriptor: [Depressive Disorder, Major] explode all trees

#92 MeSH descriptor: [Depression, Postpartum] explode all trees

#93 MeSH descriptor: [Seasonal Affective Disorder] explode all trees

#94 MeSH descriptor: [Anxiety] explode all trees

#95 MeSH descriptor: [Anxiety Disorders] explode all trees

#96 MeSH descriptor: [Stress, Psychological] explode all trees

#97 [mh ^" Mental Disorders " [mj]]

#98 MeSH descriptor: [Schizophrenia] explode all trees

#99 MeSH descriptor: [Paranoid Disorders] explode all trees

#100 schizo*

#101 psychotic*

#102 hebephreni*

#103 oligophreni*

#104 psychos*s

#105 (chronic* near mental*):ab,ti

#106 (sever* near mental):ab,ti

#107 (mental* near disorder*):ti,ab

#108 (mental* near ill*):ti,ab

#109 MeSH descriptor: [Panic] explode all trees

#110 MeSH descriptor: [Panic Disorder] explode all trees

#111 MeSH descriptor: [Phobic Disorders] explode all trees

#112 MeSH descriptor: [Combat Disorders] explode all trees

#113 MeSH descriptor: [Stress Disorders, Post-Traumatic] explode all trees

#114 MeSH descriptor: [Somatoform Disorders] explode all trees

#115 MeSH descriptor: [Hypochondriasis] explode all trees

#116 MeSH descriptor: [Hysteria] explode all trees

#117 MeSH descriptor: [Conversion Disorder] explode all trees

#118 MeSH descriptor: [Munchausen Syndrome] explode all trees

#119 MeSH descriptor: [Munchausen Syndrome by Proxy] explode all trees

#120 MeSH descriptor: [Neurasthenia] explode all trees

#121 MeSH descriptor: [Fatigue Syndrome, Chronic] explode all trees

#122 MeSH descriptor: [Obsessive-Compulsive Disorder] explode all trees

#123 MeSH descriptor: [Obsessive Behavior] explode all trees

#124 MeSH descriptor: [Personality Disorders] explode all trees

#125 MeSH descriptor: [Dissociative Disorders] explode all trees

#126 MeSH descriptor: [Compulsive Behavior] explode all trees

#127 (emotion* near disorder*):ab,ti

#128 #80 or #81 or #82 or #83 or #84 or #85 or #86 or #87 or #88 or #89 or #90 or #91 or #92 or #93 or #94 or #95 or #96 or #97 or #98 or #99 or #100 or #101 or #102 or #103 or #104 or #105 or #106 or #107 or #108 or #109 or #110 or #111 or #112 or #114 or #115 or #116 or #117 or #118 or #119 or #120 or #121 or #122 or #123 or #126 or #127 or #124 or #125

#129 #79 and #128
