# Supplementary material for: Recruitment and retention strategies in mental health trials – A systematic review
Source: PLoS One. 2018 Aug 29;13(8):e0203127. doi: 10.1371/journal.pone.0203127 (PMC6114918; doi:10.1371/journal.pone.0203127)
Supplement: S2 File — (DOCX) [file pone.0203127.s003.docx]

| Study ID | Was true randomization used for assignment of participants to treatment groups? | Was allocation to treatment groups concealed? | Were treatment groups similar at the baseline? | Were participants blind to treatment assignment? | Were those delivering treatment blind to treatment assignment? | Were outcomes assessors blind to treatment assignment? | Were treatment groups treated identically other than the intervention of interest? | Was follow up complete and if not, were differences between groups in terms of their follow up adequately described and analysed? | Were participants analysed in the groups to which they were randomized? | Were outcomes measured in the same way for treatment groups? | Were outcomes measured in a reliable way? | Was appropriate statistical analysis used? | Was the trial design appropriate, and any deviations from the standard RCT design (individual randomization, parallel groups) accounted for in the conduct and analysis of the trial? | Overall |
| --- | --- | --- | --- | --- | --- | --- | --- | --- | --- | --- | --- | --- | --- | --- |
| Man 2015 | Y | N/A | Y | N/A | N/A | Unclear | Y | N/A | Y | Y | Y | Y | Y | 8 |
| Jeste 2009 | Unclear | N/A | Y | N/A | N/A | Unclear | Y | N/A | Y | Y | Y | Y | Y | 7 |
| McLean 2014 | Y | N/A | Y | N/A | N/A | Unclear | Unclear | Y | Y | Y | Y | Y | Y | 8 |
| Dirmaier 2007 | Y | N/A | Y | Y | Unclear | Unclear | Y | N/A | Y | Y | Y | Y | Y | 9 |
| Hughes-Morley 2016 | Y | N/A | Y | N/A | Y | Unclear | Y | N/A | Y | Y | Y | Y | Y | 9 |
|  | | | | | | | | | | | | | | |
