# Supplementary material for: Recruitment and retention strategies in mental health trials – A systematic review
Source: PLoS One. 2018 Aug 29;13(8):e0203127. doi: 10.1371/journal.pone.0203127 (PMC6114918; doi:10.1371/journal.pone.0203127)
Supplement: S1 Supplementary — (DOCX) [file pone.0203127.s004.docx]

- Man 2015

A recruitment trial embedded in a large primary care research programme of two multi-centre trials. 1364 patients with depression were recruited to either original patient information materials or optimised version of the material (trial booklet). Outcome was percentage of participants randomised. 27 out of 682 patients were recruited using original patient material and 43 out of 682 patients were recruited using optimised version of the material.

- Jeste 2009

A recruitment randomised trial for a hypothetical drug trial for healthy individuals and schizophrenia patients. 128 patients with schizophrenia and 60 healthy subjects were randomised to either a multimedia educational consent procedure or general routine consent procedure. Outcomes were numbers of schizophrenia patients who were willing to participate in the hypothetical trial. A total 128 patients with schizophrenia took part in the study. 41 out of 62 patients in the multimedia group and 44 out of 66 patients were willing to participate in the hypothetical trial.

- Krusche 2014

An evaluation on 10 recruitment strategies utilised to recruit participants with a history of recurrent depression into a randomised controlled trial. Recruitment strategies include newspaper advertising, web-based advertising, advertising at exhibitions, radio advertising, advertising on buses, poster advertising, GP referrals, mental health care referrals, word of mouth, and charitable organisations referrals. Outcomes include numbers of participants who were randomised into trial through each recruitment strategy and cost effectiveness of each strategy. **Table 1** describes the recruitment information detail and the cost incurred for different strategies.

Table 1: Summary of recruitment strategy of Krusche 2014

| Strategy | Number of patients recruited | Number of patients approached | Cost per patient recruited (in GBP) |
| --- | --- | --- | --- |
| Word of mouth | 16 | 46 | 0 |
| Information from charity | 2 | 8 | 0 |
| Posters | 30 | 123 | 69 |
| Web-based adverts | 37 | 300 | 105 |
| Mental health care referral | 8 | 32 | 178 |
| Radio adverts | 26 | 412 | 241 |
| GP referral | 18 | 116 | 396 |
| Bus adverts | 2 | 4 | 571 |
| Newspaper adverts | 11 | 101 | 805 |
| Exhibition | 3 | 11 | 2562 |

- Morgan 2013

A study on internet-based recruitment to a depression prevention trial. Different online recruitment sources were used. Outcomes were numbers of participants who entered the trial. Available cost information was also collected for cost effectiveness evaluation. 755 patients were recruited via Google Advertising and 35 via Facebook Ad, with cost per patient recruited $14.71 and $19.89 (AUD) respectively.

- Rollman 2008

A comparative study between recruiting by primary care physicians’ referral to electronic medical record and recruiting by research assistants outreach in practice waiting rooms. Outcomes were numbers of patients who were enrolled in the trial through each different strategy. EMR--prompted primary care physicians referred 794 patients and 176 were recruited whereas clinical wait-room recruitment approached 8095 patients and recruited 193 subjects.

- Daley 2008

A feasibility randomised controlled trial on an exercise intervention for women with postnatal depression. 4 recruitment strategies were used in the trial, including GP referral, special care unit referral, self-referral and health visitor referral. Outcomes were numbers of patients being randomised via different strategies. 24 out of 96 patients were recruited by general practitioners; 12 out of 28 were recruited by “mother and baby unit”; 8 out of 10 were recruited from health visitors and 3 out of 4 were recruited by self-referral.

- Woolhouse 2014

A pilot randomised controlled trial using mindfulness interventions to reduce antenatal depression, anxiety and stress. 3 recruitment channels were used: recruiting at clinic waiting rooms, recruiting via mail-out from hospitals, recruiting via specialist care unit (physiotherapy and childbirth education classes). Outcomes were numbers of patients being randomised via different strategies. 14 patients were recruited at clinic waiting room, 16 were recruited via hospital mail-out, 2 were recruited at education classes.

- Debar 2009

A study that discussed recruitment for a guided self-help binge eating prevention randomised controlled trial. Recruitment strategies used includes invitation to comprehensive EDE assessment with $5 incentive for completing online questionnaire and $50 for baseline assessment, invitation to abbreviated EDE assessment with incentive of $25 for baseline assessment, and self-referral. Primary outcome was the numbers of patients who entered the randomisation of the main trial. Secondary outcomes of cost of incentives were also collected. Of the 11984 patients approached in the comprehensive EDE recruitment wave, 70 were randomised. 154 patients were recruited via the abbreviated EDE recruitment wave, in which 20810 patients were invited to take part.

- Le 2008

A study that discussed recruiting Latino women in the U.S. and women in Mexico into a multisite postpartum depression prevention trial. The study was conducted with immigrant Latinas in Washington, DC, U.S.; the other site was with women in Mexico City, Mexico. Recruitment in the U.S. used different strategies that compared outreach with potential participants by community health centre staff with recruitment by clinical research staff at a prenatal care clinic. 217 patients were recruited in the U.S. site. Primary outcome were numbers of patients who entered the randomisation. (22)

- Schlernitzauer 1998

A study that compared various strategies for recruiting elderly with bereavement-related depression in to a randomised placebo-controlled trial which tested the efficacy of nortriptyline and interpersonal psychotherapy for the acute and continuation treatment of bereavement-related depression in Pittsburgh, United States. Recruitment period lasted for 5 years. A total of 65 patients were recruited. Media advertisement was most successful strategy and recruited 54% subjects (n=35). Other strategies used included friend or acquaintance, obituary letter, psychiatric referral, and so on.

- Hughes-Morley 2016

A recruitment randomised trial for the EQUIP host trial – a clustered RCT of a new user led training package to increase user and carer involvement in care planning for patients with a diagnosis of severe mental illness under community mental health teams. Patients with different cluster pairs were randomised to either invitation with patient and public involvement research (PPIR) invitation leaflet or ordinary recruitment procedure. 216 out of 5382 patients were recruited in the PPIR group and 148 out of 2800 were recruited in the ordinary recruitment group. Telephone follow up of non-responders recruited a total of 221 patients. However no significant difference did PPIR make to improve recruitment.

- Dirmaier 2007

A randomised study with 2×2 factorial design that investigated monetary incentives and shortening the questionnaire in relation to response rates in a mailed follow-up survey 1 year after psychotherapeutic treatment. Partial nonresponse and self-report of treatment outcome were also assessed. 3825 patients were randomised to (1) receiving prepaid small bill incentive or none; and (2) getting abridged or normal questionnaire. It showed that response rates were significantly increased by 7.3% (95%CI 2.6 to 11.9%) when using incentives, and a 3.7% (95%CI 0.9 to 8.3%) increase when using a short version of questionnaire.

- McLean 2014

A randomised study that investigates the effect of pre-notification or envelop teaser on response rates in a bulimia nervosa mental health literacy survey. A 2 (pre-notification present; absent) by 2 (teaser present; absent) design was used. Questionnaires were mailed to 3010 adults, and significantly higher response rates were found for the use of pre-notification.
